# Role of Type I Interferon (IFN) in the Respiratory Syncytial Virus (RSV) Immune Response and Disease Severity

**DOI:** 10.3389/fimmu.2019.00566

**Published:** 2019-03-26

**Authors:** Diego R. Hijano, Luan D. Vu, Lawrence M. Kauvar, Ralph A. Tripp, Fernando P. Polack, Stephania A. Cormier

**Affiliations:** ^1^Department of Infectious Diseases, St Jude Children's Research Hospital, Memphis, TN, United States; ^2^Department of Biological Sciences, Louisiana State University and School of Veterinary Medicine, Baton Rouge, LA, United States; ^3^Department of Comparative Biomedical Sciences, Louisiana State University School of Veterinary Medicine, Baton Rouge, LA, United States; ^4^Trellis Bioscience, LLC, Redwood City, CA, United States; ^5^Department of Infectious Disease, University of Georgia, Athens, GA, United States; ^6^Fundacion INFANT, Buenos Aires, Argentina

**Keywords:** infant immunity, respiratory syncytial virus, type I interferons, human, mouse, vaccine

## Abstract

Respiratory syncytial virus (RSV) is the most common cause of lower respiratory tract disease in children <2 years of age. Increased morbidity and mortality have been reported in high-risk patients, such as premature infants, patients with cardiac disease, and severely immune compromised patients. Severe disease is associated with the virulence of the virus as well as host factors specifically including the innate immune response. The role of type I interferons (IFNs) in the response to RSV infection is important in regulating the rate of virus clearance and in directing the character of the immune response, which is normally associated with protection and less severe disease. Two RSV non-structural proteins, NS1 and NS2, as well as the envelope G glycoprotein are known to suppress type I IFN production and a robust type I IFN response to RSV does not occur in human infants or neonatal mouse models of RSV infection. Additionally, presence of type I IFNs are associated with mild symptoms in infants and administration of IFN-α prior to infection of neonatal mice with RSV reduces immunopathology. This evidence has driven RSV prophylaxis and therapeutic efforts to consider strategies for enhancing type I IFN production.

## Introduction

Respiratory syncytial virus (RSV) is a common cause of lower respiratory tract disease in infants and young children (1–3). Although 30–70% of infants develop bronchiolitis upon primary RSV infection, only 1–3% are hospitalized (4). Despite this heterogeneous course of disease, the global burden of RSV disease is estimated at 64 million cases and 160,000 deaths annually (5, 6). Increased morbidity and mortality have been reported in high-risk patients, such as premature infants, infants with cardiac disease, and severely immuno-compromised patients (7–9). Moreover, the consequences of severe RSV infection are long lasting and constitute a risk factor for childhood asthma and bronchiolitis (10–14). The elderly and immune compromised also suffer from RSV, particularly those with prior pulmonary problems (15). Notwithstanding the advances in our understanding of the immune response to RSV and the recently determined high resolution structures of the two major immunogenic viral proteins, the RSV F and G proteins, we still lack adequate therapeutics as well as a safe, robust, and effective vaccine (16).

Both viral and host immune factors have been implicated in severe infections (17–20). RSV is an orthopneumovirus in the Paramyxoviridae family (21, 22). The RNA genome contains 10 genes encoding 11 proteins. The envelope of the virus is formed by the matrix (M) protein, the small hydrophobic (SH) protein, and two abundant, glycosylated surface proteins: the fusion (F) and attachment (G) proteins. The G and F proteins control the initial phases of infection (23, 24). The G protein is composed of three epitope regions identified by murine monoclonal antibodies: mostly invariant epitopes in the central conserved domain (CCD); group-specific epitopes (subtype A or B); and strain-specific epitopes in the C-terminal hypervariable region of the G protein ectodomain (25, 26). The two antigenically distinct subtypes, A and B, can co-circulate during the same epidemic season (27–29). The clinical impact of different subtypes likely contributes to different disease severity. While the F protein has historically been the major target for antiviral and vaccine development, both G and F proteins are naturally targeted by neutralizing antibodies induced by infection (23, 24, 30–33). The two non-structural proteins, NS1 and NS2, suppress IFN production (34–36), with NS1 known to bind RIG-I within the cytoplasm of host cells thereby abrogating the signal transmitted via MAVS (2). Further, the G protein also impedes IFN-α expression through the interaction of the CX3C chemokine-like motif in G, which interacts with CX3CR1 and impairs the immune response to RSV. Infection with an RSV strain that lacks the CX3C motif (mimic of the human chemokine called fractalkine or CX3CL1) or treatment with an anti-G monoclonal antibody (MAb) that blocks binding to CX3CR1 result in increased levels of type I/III IFN (37).

The fractalkine receptor, CX3CR1, is expressed on human plasmacytoid dendritic cells (pDCs) and epithelial cells (37–39). The former are specialized immune cells that infiltrate the lung to produce large amounts of type I IFN in response to viral infection (40, 41).

The link between RSV G protein and type I IFN expression is well established (42–44) with details elucidated that include TLR4 signaling and SOCS3 regulation of type I IFN (45–50). For example, the RSV G protein contributes to immune evasion by modifying host cytokine and chemokine responses whose expression is negatively regulated by suppressor of cytokine signaling (SOCS) proteins (48). SOCS1 and SOCS3 are closely related and well characterized members of the family acting through the JAK/STAT pathway to regulate cytokine expression via a kinase inhibitory region (51). SOCS1 and SOCS3 are downstream from toll like receptors (TLR) and can indirectly regulate them (52). Specifically, SOCS3 induction by TLR is dependent on Myd88 (52). SOCS1 and SOCS3 strongly suppress TLR7-mediated type I IFN production by binding IFN regulatory factor 7 (53). In addition, SOCS1 modulates TIRAP which is downstream of TLR1/2, TLR2/6 and TLR4 but not TLR9 (51). It has been shown that SOCS1 and SOCS3 regulate type I IFN in normal fully-differentiated human bronchial epithelial (NHBE) cells, with the pathway including interferon-regulatory factor (IRF)-3 activation and nuclear translocation (48). Further, interferon-stimulated gene (ISG)-15 expression is altered very early after infection and RSV infection has been shown to upregulate SOCS 1 and SOCS 3 in epithelial cells (46). NHBE cells infected with an RSV mutant virus lacking the G gene have distinct responses as compared to wild-type RSV (30). Notably, RSV mutant strains without secreted G induced less CCL2 and CCL5 with no apparent lung disease in mice. Interestingly, mice developed good antibody responses despite the attenuated infection (54). These findings suggest that RSV surface proteins signal through multiple pathways, and this may be an important means of reducing anti-viral type I IFN expression, thereby promoting virus replication.

Of interest, RSV does not induce robust, long term immunity and people may be repeatedly infected with the same and different strains of RSV (55, 56). These finding are particularly relevant to the multiple failed RSV vaccine trails to date, including the original formalin inactivated RSV (FI-RSV) vaccine as well as more recent subunit and live attenuated vaccines. The deficient response to both natural and artificial exposure to RSV antigens in human represents a barrier to the development of novel therapeutic or preventive strategies (57–64). Further, the immune response to both primary and repeat infections with RSV needs further study to better understand short- and long-term immunity. More detailed characterization of the response of healthy adults as compared to the elderly and to infants is also needed. The importance of elucidating the host response to RSV infection is underscored by recent clinical evaluation of prophylaxis with the anti-F protein monoclonal antibody (mAb) palivizumab in healthy preterm infants. In this single-blind, randomized, placebo-controlled trial, suppression of RSV replication did not have a major effect on reducing the RSV-associated asthma incidence at age 6 years, suggesting that other factors besides viral load contribute to the clinical severity (11, 65).

Type I IFNs are a group of related proteins that help regulate the activity of the immune system. The mammalian types are named IFN-α (alpha), IFN-β (beta), IFN-κ (kappa), IFN-δ (delta), IFN-ε (epsilon), IFN-τ (tau), IFN-ω (omega), and IFN-ζ (zeta) (66, 67). IFN-α has 13 different subtypes in humans (α1/13; α2; α4; α5; α6; α7; α8; α10; α14; α16; α17; α21) (68) and is primarily produced by pDCs, while IFN-β is produced largely by fibroblasts; both have antiviral activity that is an important component of the innate immune response. Quantitative and qualitative differences in gene expression have been observed, with type I IFN being notably absent in the RSV infected cells (69). This result is consistent with results from the INFANT study, conducted by Argentine doctors to investigate the causes of respiratory diseases that seriously affect children such as RSV associated asthma and bronchiolitis, and pneumonia and influenza virus infection. In the INFANT study, RSV infection failed to induce a robust type I IFN response in the nasal mucosa of infants even when co-infected with influenza, which normally induces a robust response (70). Intriguingly, neonatal mouse models of RSV infection recapitulate these data from humans. Specifically, neonatal mice infected with RSV fail to induce a type I IFN response to RSV in contrast to adult mice infected with RSV (71). Furthermore, as compared to non-treated controls, administration of IFN-α during infection of the neonate enhances the immune response to RSV infection 5 weeks later and prevents Th2 biased immune responses (including perivascular inflammation and mucus production) and airway hyperreactivity (71). Notably, studies examining human cord blood-derived pDCs exposed to RSV showed reduced type I IFN production when compared to vehicle control or left unstimulated (40). These recent correlations between type I IFN responses and RSV disease severity in infants merit further investigation. Here, we review the mechanism surrounding RSV and type I IFN production in humans and mouse models and discuss its implications for development of therapeutics and vaccines.

### IFN Biology and RSV Disease

Human IFNs are classified as type I (IFN-I), type II (IFN-II), or type III (IFN-III) with each class binding to specific receptors. All type I IFNs bind to a specific cell surface receptor complex known as the IFN-α receptor (IFNAR) that consists of IFNAR1 and IFNAR2 chains (72). The ability to produce and respond to IFN-I is distributed in a wide variety of cells. This confers several autocrine and paracrine effects that have been extensively characterized, mainly in viral infections. IFN-I signaling is mediated through a common cell surface receptor, the IFN-I receptor (IFNAR), signaling through the JAK-STAT cascade leading to transcriptional upregulation of the IFN-ISGs. The IFN-II family is represented by a single gene product, IFN-γ, and is mainly produced by T lymphocytes and natural killer (NK) cells. The associated receptor (IFNGR) regulates several cell functions related to host defense to intracellular pathogens. IFN-λ comprises four subtypes: IFN-λ1, IFN-λ2, IFN-λ3, and IFN-λ4. The members of this IFN-III family interact through a unique receptor, the IFN-λ receptor (IFN-λR). It has been shown that IFN systems differ in terms of tissue distribution of their receptors (73, 74). While IFN-α/β systems are more prominent on endothelial cells, they are expressed on all cells. On the other hand, IFN-λ expression is more restricted occurring predominantly on epithelial cells of the intestines and lungs (73). RSV infection induces high expression levels of IFN-λ 1–3 in the lungs, and these have been associated with more severe disease in children (75).

Type I and III IFNs are induced in virtually all cell types upon recognition of viral proteins by cytoplasmic and endosomal receptors (67, 68). IFN induction by RSV involves the recognition of RSV by TLRs which activate innate and acquired immunity (47, 49, 76–78). Leukocytes express several TLRs, including TLR2, TLR6, TLR3, TLR4, and TLR7 (79). Using knockout mice, TLR2 and TLR6 signaling in leukocytes has been shown to activate innate immunity against RSV by promoting TNF-α (tumor necrosis factor), IL-6 (interleukin-6), CCL2 (monocyte chemoattractant protein 1), and CCL5 (RANTES) (80). TLR4 was shown to also contribute to cytokine activation, and TLR2 and TLR6 activation was shown to be important for controlling viral replication *in vivo* in mice (81). TLR2 interactions with RSV promoted neutrophil migration and dendritic cell activation within the lung. TLR3 has been associated with more severe disease in mice models (82).

TLR4 is upregulated by RSV F protein interaction with TLR4 (76, 77). RSV G protein reduced TLR4 activity to baseline levels even in the presence of LPS (lipopolysaccharide), a strong stimulus, as assayed using a luciferase reporter construct for TLR4 signaling (76). As previously noted, RSV infection of normal human bronchoepithelial cells has been shown to modulate expression of SOCS, an effect mediated by G protein, leading to inhibition of type I IFN and ISG15 expression (48). These findings suggest that RSV surface proteins signal through multiple TLRs, and that enhanced expression and activation of type I IFNs may promote viral replication. Accordingly, IFN-α has been considered as an adjuvant for RSV vaccines as it is known to promote the activation and survival of virus-specific T cells (83).

The role of type I IFN in RSV infection, shedding, and disease severity in humans has been a subject of interest for decades (84, 85). While early studies struggled to identify a role for type I IFN in RSV disease (84–88), novel findings in recent years implicate type I IFN as determinants of RSV pathogenesis and immune responses (40, 41, 89, 90). RSV is a poor inducer of IFN and as a consequence, these IFNs and related cytokines have been speculated to have a limited role in the host defense against viral infection (84, 85, 87, 88). In fact, most hypotheses for RSV disease susceptibility in infants have been based on unique structural respiratory factors such as smaller airway size, lack of interalveolar pores and channels and different innervation patterns, inflammatory responses, and Th2 polarization of the adaptive immune response (78, 91, 92). Reconsideration of this bias is needed. Unlike the case in infants and children infected with influenza virus, IFN levels were undetectable or low in nasal secretions of infants and young children with RSV lower respiratory tract illness and did not correlate with resolution of clinical signs (84, 85). In a more recent study of infants in Argentina, type I IFN was detected more frequently in those infected with influenza A virus than in those infected with RSV or hMPV (93). RSV infected infants hospitalized with bronchiolitis displayed low, intermittent concentrations of IFN-α in respiratory secretions (87). No significant correlation was seen between these low respiratory IFN levels and RSV shedding (88). In human macrophages and peripheral blood mononuclear cells, RSV infection also induced minimal IFN activity and elicited no detectable transcription of IFN-α or IFN-β gene products (86), which is consistent with low IFN-α production in monocyte cultures from young infants (40).

Intriguingly, RSV-induced IFN-α expression by primary pDC collected from older children (from 1 to 5-year-olds) was notably higher than that of healthy full-term infant counterparts suggesting expression may be linked to age of the patient. Likewise, higher IFN-α expression was detected in primary pDCs obtained from healthy adults (40). Age at the time of initial infection is an important predictive factor for disease severity (94, 95). Cohort studies demonstrated that young infants (<6 months of age at initial infection) are at greater risk for severe disease than older infants (96, 97). Furthermore, long-term consequences of RSV infection, such as development of asthma, are closely associated with severity of infection (10, 13). Extrapolation of response to vaccines or therapeutics in adults to those in young infants is thus highly problematic.

While clear linkage between IFN expression and RSV infection in humans has been elusive, a factor that needs further study is the prolonged incubation period of RSV disease in infants for whom the mean time from infection to symptoms is 4–6 days (87) in sharp contrast to the considerably shorter incubation period for influenza virus (average of 2 days). Type I IFN levels peak early after infection, and therefore sampling of respiratory secretions after symptoms appear may be too late to detect its antiviral effects for infants infected with RSV (84, 85, 93). Support for a function of type I IFNs in RSV pathogenesis is also growing from analysis of developmental innate immune mechanisms associated with poor type I IFN responses in newborn and young infants. For instance, and as mentioned above, RSV-induced IFN-α production appears to be primarily mediated by pDC, (40, 41). Indeed, compared to adult pDC production of type I IFN during RSV infection is substantially impaired in infants when disease is particularly severe (40, 90). Impairment in infants is explained by deficits either in MAVS or RIG-I at the post-translational level or by signaling events downstream of MAVS (40).

Additional evidence supporting a role for type I IFN in RSV infection and illness is the strong inhibition of IFN induction and signaling mediated by the two earliest genes transcribed among the 11 RSV gene products, NS1 and NS2 (89). NS1 and NS2 have been postulated to have various roles in RSV pathogenesis, generally linked to their anti-IFN activity. In addition to antagonizing type I IFN, NS1, and NS2 may negatively modulate dendritic cell maturation, affect Th17 lymphocyte proliferation, and promote Th2 polarization (35, 98–105). Deletion of anti-IFN proteins NS1 and NS2 in RSV live vaccines is responsible for attenuated phenotypes (89).

In the era prior to availability of antibodies against RSV, topical administration of recombinant IFN-α-2a accelerated control of upper respiratory tract symptoms during RSV infection in a randomized, double-blinded trial while not affecting duration or magnitude of viral shedding (106). This early result is of interest in the context of a more recent study of nasal epithelial cells from children with wheeze and/or atopy that showed reduced IFN-β in the nasal swabs in response to RSV infection, which was associated with increased viral shedding (107). However, consistent with other successful immunotherapies, this regimen elicited adverse effects and severity of those effects were dose-dependent (108). Common side effects due to IFN-α include flu-like symptoms, pulmonary toxicity (109), gastrointestinal symptoms (110), and neurotoxicity (111). Lethal toxicities associated with IFN-α regimen are rare and severe toxicities due to IFN-α are manageable if recognized expeditiously (112, 113). Importantly, IFN-α therapy in children (114) and infants with RSV-induced bronchiolitis (115) is generally safe and well tolerated. However, caution is still warranted in use of recombinant IFN-α in the context of an RSV infection, due to the side effects mentioned above.

It is also possible that antiviral agents may benefit from restoring natural type I IFN responses, which may lead to faster clearance of the virus. Two studies using healthy adult volunteers experimentally infected with RSV and treated with antivirals showed that rapid RSV clearance was related to reduced disease (116, 117). Similarly, a higher RSV load was linked to an increased risk for severe bronchiolitis in a large multicenter trial in the United States (28). None of these studies have attempted to define the mechanism by which higher viral load contributes to disease severity. In that regard, a study in infants with RSV bronchiolitis that described an association between viral load and disease severity (length of hospital stay) is of interest since a correlation was also noted with relative expression of ISG-56 (118). Finally, additional evidence for the role of type I IFN in disease severity comes from two studies of rare loss-of-function variants in *IFIH1* (which encodes a RIG-I-like receptor involved in the sensing of viral RNA); the variants result in defective innate recognition of RNA viruses preventing the activation of an efficient antiviral IFN response. These rare but serious immunodeficiencies lead to extreme susceptibility to RSV and other respiratory viruses (119, 120).

### Responses in Mice

Mice provide a semi-permissive model for human RSV and while attempts to adapt a strain to this model have repeatedly failed (121) data from numerous laboratories demonstrate similarities in age related immune responses between humans and neonatal mice. Since, our current understanding of the features that contribute to severe RSV disease in infants is tied to our understanding of developmental immunity during the first year of life, the neonatal mouse model of RSV infection is a helpful tool (122–124). Numerous studies utilizing mouse models of RSV infection have revealed a bias toward a T helper type 2 (Th2) cytokine response when mice are initially infected as neonates as compared to adults (71, 125–128). Upon reinfection, mice initially infected as neonates mount significantly greater Th2 responses as compared to mice initially infected as adults (126). This skewed Th2 response upon reinfection is associated with lung dysfunction (lung eosinophilia, increased mucus production, and air hyperresponsiveness) (126, 127, 129). Such responses mirror observations made in infants with severe RSV disease (130–132). Production of type I IFN by pDC during RSV infection of the neonate mouse, as in humans, is considerably impaired. However, both pDC number and production of type I IFN in response to RSV increase with age; adult mice recruit substantially higher numbers of pDCs to the lungs after RSV infection when compared to those of same age that are not infected and to neonatal mice infected with RSV (71). A single dose of IFN-α or adoptive transfer of adult-derived pDCs (capable of mounting a type I IFN response), prior to a primary RSV infection, substantially impedes the Th2-biased immunopathology observed during reinfection (71). A related strategy to revert poor outcomes associated with RSV infection in neonatal mice has been administering Flt3 ligand to neonates before RSV infection (133). Ftl3 ligand is a growth factor that stimulates the proliferation of hematopoietic cells that triggers expansion of cDCs and pDCs in human cord blood and strongly promotes IFN-α production by pDCs in response to viral exposure (134, 135). This treatment has led to increased lung DC numbers and reconditioning of the type I IFN pathway toward Th1-mediated immunity. In addition, these mice were protected from exacerbated airway disease upon adult re-exposure to RSV (133).

Treating mice with neutralizing mAbs against the RSV G protein reduced G protein-mediated lung inflammation. Specifically, TRL3D3, a human mAb against the G protein CCD, enhanced IFN responses, decreased airway inflammation, and improved lung function upon secondary infection, whereas mice treated with an anti-F mAb (palivizumab) had less IFN than mock infected mice (30, 33). Since RSV infection is inhibited by IFN-induced transmembrane proteins (71, 117), the impact of counteracting the G protein's suppressive effect on IFN production likely also contributes to the antiviral effect of such mAbs. Consistent with these results, intranasal IFN-α administration in neonatal mice prior to RSV infection appreciably reduced RSV viral load in both nasal associated lymphoid tissue and lungs when compared to age-matched controls (136).

**Figure 1:**
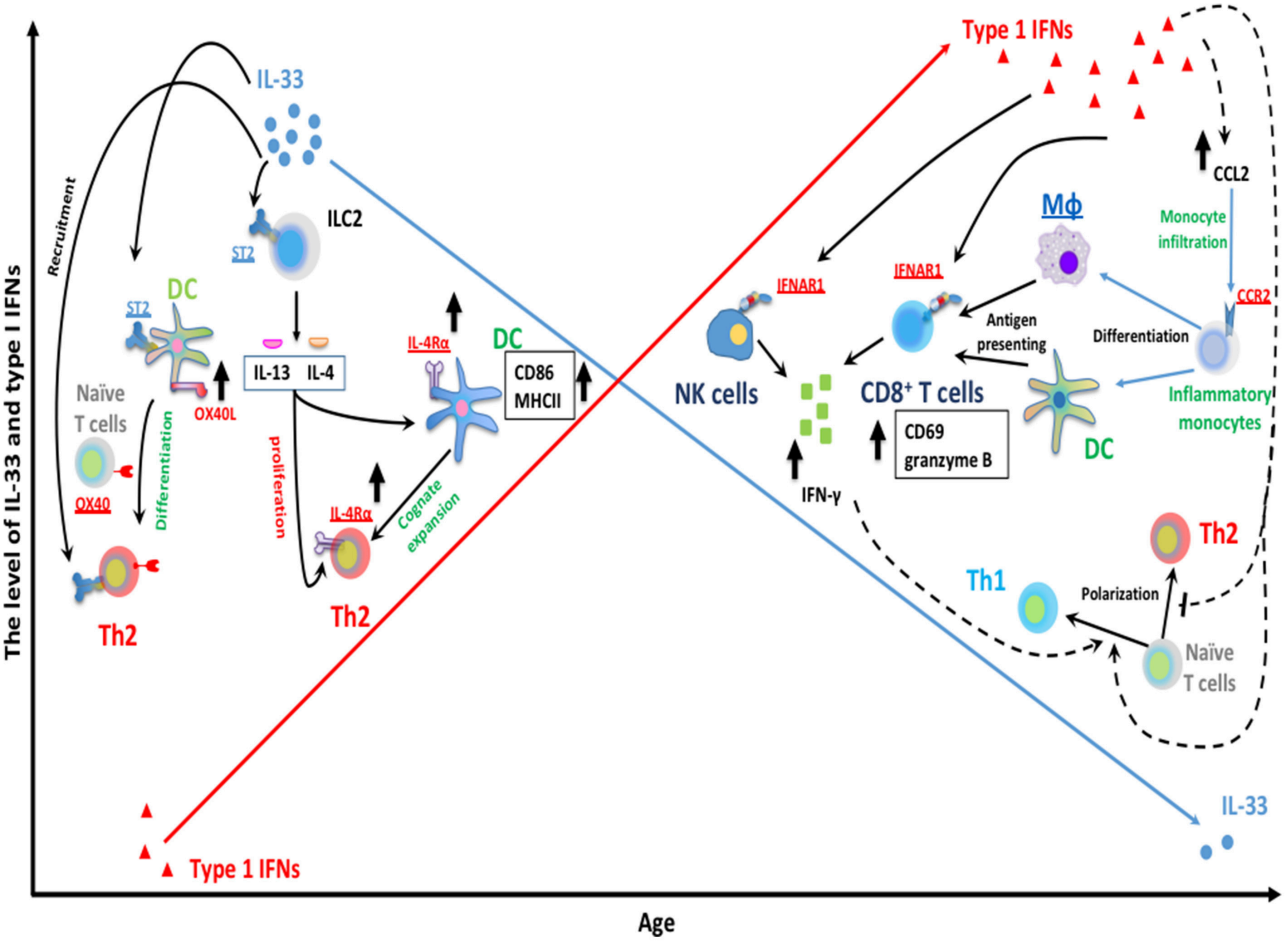
Age-dependent differences RSV-mediated immune pathogenesis. The expression of RSV-induced IFNα is limited during infantile RSV infection and progressively increases with age. Type I IFNs are capable of suppressing Th2 development and promoting type 1 immunity. Type I IFNs have been implicated in the regulation of NK and CD8^+^ T cells functionality. Type I IFNs can elicit the activation of cytotoxic IFN-γ^+^ CD8^+^ T cells by enhancing the recruitment of inflammatory myeloid cells into infected lungs. These infiltrating myeloid cells then differentiate into macrophage and DC, and acquire antigen presenting ability, subsequently activate CD8^+^ T cells and trigger CD8^+^ T cells IFN-γ production. In addition, type I IFNs can also act directly on CD8^+^ T cells and NK cells by targeting its receptor IFNAR1 on membrane of CD8^+^ T cells and NK cells. This results in the production of IFNγ, a key mediator for type 1 immunity, which presumably favors Th1 polarization from naïve CD4^+^ T cells. In contrast with IFN-α, IL-33 has been implicated in the induction of Th2-biased immune pathogenesis during neonatal RSV infection. A large amount of IL-33 is rapidly released following RSV infection in neonatal but not adult mice. IL-33 can elicit ILC2-mediated IL-13/IL-4 production through its cellular receptor ST2 on ILC2. ILC2-derived IL13/IL-4 then can facilitate cognate expansion of Th2 by upregulating the expression of Th2 costimulatory molecules (CD86 and MHCII) on DC. ILC2-derived IL-4 also promotes the proliferation of Th2 cells. It involves the upregulation of IL4Rα on both DC and Th2 cells. Similarly, IL-33 can promote Th2 polarization from naïve CD4^+^ T cells by targeting DC via ST2 receptors on DC and then enhance the expression of OX40L on DC (ligand for cellular receptor OX40 on naïve T cells).

Interestingly, while the IFN-α response to RSV progressively increases with age (40, 136); another cytokine IL33, an alarmin cytokine, decreases with age (126). Recent work has demonstrated that IL-33 is significantly greater in neonatal compared to adult mice during RSV infection. IL-33 signaling in the neonatal mouse model of RSV has been shown to induce RSV immunopathogenesis including Th2 bias (126). Elevations in IL-33 are inversely correlated with age at RSV infection (126) and severity of RSV infantile disease has been associated with elevated levels of respiratory IL-33 and polymorphisms within *ST2*, the receptor for IL-33, (137). IL-33 promotes Th2 responses via multiple signaling pathways that are summarized in Figure 1. Similarly, intranasal instillation of IL-33 significantly impaired the production of IFN-α/λ in the BALF and reduced the expression of IFN-stimulated genes in the lung following PVM infection (138). Table 1 summarizes the significant advances in the role of age-dependent differences in various immune and non-immune cells related to the immune pathogenesis of RSV infection in infants. Figure 1 highlights age-dependent differences in RSV-mediated immune pathogenesis.

**Table 1:** Differences in immunological responses toward RSV in the respiratory tract.

**Respiratory immune responses**	**Adult mice**	**Neonatal mice**	**Human infants**
**IFNα UPSTREAM SIGNALING**			
Respiratory/pulmonary pDC	+++ (71, 139)	+ (71, 139)	older (≥4 months) infants had fewer BAL pDCs than younger (<4 months) (140)
IFN-α	+++ (71, 133, 139)	+ [(71, 139), Remot, 2016 #432]	IFN-α production by primary pDC collected from healthy term infants is lower than older children (from 1-year to 5-year-olds) (40)
**IFNα MEDIATED IgA-PRODUCTION OF B CELLS**			
Nasal associated lymphoid tissue—B cells	+++ (136)	++ (136)	
Respiratory IgA	+++ (136, 141)	+ (136)	IgA levels in nasal aspirates are lower in younger infants (4–8 months) compared to older infants and young children (9–21 months) during RSV infection (142, 143)
**OTHER IMMUNE MEDIATORS**			
CD103^+^/CD11b^+^ DC	+: CD103^+^/CD11b^+^ ratio (144)	+++: CD103^+^/CD11b^+^ ratio (144)	
	+++: CD80 and CD86 (144)	+: CD80 and CD86 (144)	
	+: OX40L expression (145)	+++: OX40L expression (145)	
CD4^+^ T cells	Th1 responses >Th2 responses (126, 146)	Th2 responses >Th1 responses (126, 146)	Th2 responses > Th1 responses (131, 132, 147) (Cormier SA, Unpublished Data)
CD8^+^ T cells	+++: IFN-γ producing (148)	+: IFN-γ producing (148)	+++ (149) and Tc2 responses > Tc1 responses (Cormier SA, Unpublished Data)

*(+, low; +++, high)*.

### Implications for RSV Vaccines and Therapeutic Agents

Current RSV vaccine candidates seek to induce high levels of RSV-specific serum neutralizing antibodies, which are associated with reduced RSV-related hospitalization rates. However, serum neutralizing antibodies may not be sufficient to prevent infection and/or induce protective responses. This feature of RSV biology was exemplified by the antibody responses induced to the FI-RSV vaccine in the 1960's, which elicited lower avidity, non-protective antibodies as compared to those that develop after natural RSV infection (150). Furthermore, mucosal antibodies have been shown to correlate better with RSV protection than serum antibodies in both infants and adults (151–153).

The majority of vaccine efforts to date have focused on the RSV F protein, based on the assumption that reducing RSV load will reduce or eliminate disease. While mAbs against RSV F protein (palivizumab) given to premature infants (at or before 35 weeks) do help to protect children with certain lung or heart conditions who are at high risk for severe RSV disease, such treatment does not fully protect from disease. Further, in a recent study of viral burden in healthy full-term infants (<70 days old), nearly a third experienced a multi-log rebound in viral load at around 2 weeks after onset of symptoms (154). Since viral load had declined by several orders of magnitude by that point, the most likely cause was mutational escape which is a well characterized response to anti-F protein mAbs (155).

In short, the role of RSV viral load as a driver for severity of infection remains controversial. On the one hand, quantitative RT-PCR correlation with disease severity in patients showed that viral load was associated with disease severity in younger patients although not in older patients (63). For patients intubated due to respiratory distress, RSV infection resulted in higher viral load than those not intubated, and higher viral loads were associated with longer hospitalization (156). In the adult human RSV challenge model, virus replication is inversely correlated with the level of nasal secretory neutralizing antibody prior to infection (157). Higher nasal immunoglobulin (Ig) A predicts lower infectivity and lower measures of viral replication (151) and low RSV-specific nasal IgA is an independent significant risk factor for RSV infection (158). On the other hand, several groups have failed to find an association of higher viral load in nasopharyngeal lavage (159) or nasal aspirates with either length of hospitalization, duration of oxygen supplement or severe bronchiolitis in either infants (160) or children (161).

The picture that is emerging is that primary reduction in viral load is useful, but not sufficient, to reduce the clinically relevant pathology. Accordingly, a combination of an anti-viral agent with an agent that reduces the RSV induced alteration in the innate immune response is the most likely route to improved outcomes. Targeting the F protein addresses the first issue. Targeting the G protein addresses the second issue; since anti-G protein mAbs also have potent antiviral activity, targeting the G protein alone may be sufficient to achieve both goals.

The optimal type of RSV vaccine employed, i.e., RSV F and/or G protein, will likely be dependent on the host target population (162, 163), with four groups being of interest: (1) infants and young children, (2) adults, (3) the elderly, and (4) pregnant woman. Immunization schedule (prime/boost) and the specific platform for delivery of the vaccine are also likely to be important (162, 164, 165). Consequently, there are a spectrum of RSV vaccines being tested that include live-attenuated and chimeric virus, purified F protein (including variants engineered to present predominantly the pre-fusion conformation), particle and vector-based presentations of the antigen(s) (165). For example, RSV F protein particle-based (Novovax) (166, 167) and RSV F subunit (GSK; NIH) vaccines are being evaluated for use in pregnant mothers, while RSV F protein particle-based (Novovax; Mucosis) and live-attenuated vaccines such as RSV deletion mutant vaccines, e.g., ΔM2-2 and ΔNS2 constructs (Sanofi; NIH) are being targeted for the pediatric population with potential extension to older children and young adults (168). An important caveat for using live vaccines is the need to prevent transmission to the immune compromised, or those with reduced or waning immunity. An additional issue for vaccinating infants and young children is that the vaccine needs to balance safety (higher attenuation) and efficacy (lower attenuation). A promising recent study of an RSV vaccine candidate having a deletion of the M2-2 coding sequence showed downregulation of viral replication and upregulation of transcription and antigen synthesis (169). For healthy older adults, several RSV vaccine candidates are being considered, including vector-based platforms such as VXA-RSV F oral (Vaxart) and Ad26.RSV.preF (Janssen) (168). Given the high transmissibility of RSV, even a safe and effective vaccine will likely leave gaps in protection for high-risk, very young infants. Vaccinating pregnant women has become an area of high interest to induce passive protection in the infant by generating high maternal antibody titers.

Antibodies directed to dominant antigenic sites on the F protein have variable neutralization capacities with the most potent neutralization epitopes associated with the pre-F conformation (170–174). Stabilized F protein antigen in both pre- and post-fusion morphology are being explored (31, 172, 175, 176). The typical benchmark is achieving a protective titer at a defined time point, but the time course of increase in antibody titer is also an important parameter, which will likely differ according to vaccine type and composition. The RSV G protein is also an antigenic target for neutralizing antibodies, but despite this fact, the G protein has not usually been considered as a RSV vaccine candidate because of its variability across RSV strains (175–177). However, with the recent discovery of the G protein structure (29, 32), and the known role of the G protein oligomer on the virus surface vs. its monomeric secreted form (54, 178), there has been reinvigorated interest in its potential as a RSV vaccine candidate.

Passive transfer of antibodies is protective against severe RSV infection using polyclonal or monoclonal antibodies (mAbs; RSV-IVIG, palivizumab) (179) The ratio of antibody transfer and decay kinetics is considered a principal parameter to measure protection. More recent versions of mAbs have become available with improved antibody transfer and decay kinetics such as MEDI-8897 which is optimized from the human antibody D25 that targets RSV pre-F protein (24, 170, 172, 180, 181). This type of treatment potentially offers novel immunotherapeutic strategies to bridge gaps with RSV vaccine candidates.

Many studies indicate that certain cytokines can mediate strong vaccine responses associated with a good outcome. For example, IFN-α2b is an FDA-approved therapy for adjuvant treatment of patients with certain cancers (182) and hepatitis C (183). Of particular interest is the recent demonstration that administration to neonatal mice of IFN-α prior to RSV infection increased RSV specific IgA production in nasal washes when compared to age matched controls (136). Furthermore, IgA levels became comparable to those of adult mice infected with RSV (136). In addition, IFN-α induced expression of B cell activating factor (BAFF) in nasal associated lymphoid tissue (NALT) (136). BAFF, a B cell survival factor and mediator of B cell activation and class switching, and APRIL, a TNF ligand family member that shares receptors with BAFF, regulate B cell survival, proliferation and differentiation. Gene expression analysis from NALT and lung homogenates further support a role for IFN-α in regulating granulocyte migration and neutrophil-mediated immunity (136).

Comparative studies of genetic background of mice has shown diverse influences on Th cell differentiation by controlling the capacity for IL-2-induced IL-4 production by naive CD4+ T cells. BALB/c mice are Th2-prone, while C57BL/6 mice are Th1-prone (28, 184–186). Notably, type I IFN pathways are reconditioned in neonatal BALB/c mice after RSV infection as lung dendritic cells (DC) numbers increase; the associated shift toward a Th1 response protected the mice from exacerbated airway disease (187). Adult mice produce considerably higher levels of type I IFNs in response to RSV than do neonatal mice. Finally, recent studies have implicated the type III IFN-λ as being significant for mucosal antiviral immune responses to RSV infection (41, 65).

Since SOCS-1 and SOCS-3 negatively regulate the IFN-induced signal cascade, and NS1, NS2, and G protein inhibit the type I IFN response, any of these viral proteins may prove to be useful targets to induce a more effective innate immune response (45, 50). Understanding how these viral proteins modify host immune responses is thus crucial to the development of effective countermeasures. Although no animal model perfectly mimics the human response, the mouse offers a far greater set of tools for analyzing the immune system than other popular models, such as the cotton rat, and the mouse has for that reason become the nearly exclusive model for studies on RSV and the host immune response.

### Clinical Implications

Over the past decade, targeting the F protein has repeatedly produced disappointing clinical results. In particular, agents targeting the F protein have not been proven effective post-infection. This is not only problematic for the multiple populations in need of treatment but also for vaccines since healthy full term infants (<70 days old) experienced a significant rebound in viral load at around 2 weeks after onset of symptoms in nearly a third of the study population (154). Moreover, palivizumab is only approved for prophylaxis in premature infants and those at high risk for severe RSV disease. Retrospective analysis of samples from the clinical trials leading to approval of this drug revealed a striking skewing of TLR4 polymorphisms (188). Mutations that interfere with function of this key innate immune system receptor have an incidence in the general population of ~10%, but 90% of the high-risk premature birth infants had a TLR4 mutation. This striking result has been replicated (78). As described above, the RSV F protein stimulates TLR4, while the G protein suppresses this signaling pathway (48). In the premature birth population, antibody mediated removal of the TLR4 stimulus should not impact the overall response since the pathway is already suppressed genetically. In the broader population, however, removal of that beneficial stimulus may contribute to the lower observed efficacy compared to what was expected.

In light of these empirical failures and the improved understanding of RSV disease mechanisms, interest has increased in the role of the other major viral envelope protein, the RSV G protein, on viral entry, on viral neutralization, and most critically on RSV-mediated pathology (33). In mouse pDCs, mutating the G protein CCD prevented suppression of IFN-α attributable to the G protein; the Fab of a murine mAb against this region of the G protein was nearly as effective as the mutation (39).

Human mAbs targeting the CCD of RSV G protein (189) have recently been compared to anti-F mAbs, as both prophylactic and therapeutic treatment in BALB/c mice. The results showed that targeting the G protein was more effective for reducing viral load, leukocyte infiltration, and pro-inflammatory cytokine expression in cell-free bronchial alveolar lavage (BAL) supernatants (190). These results are consistent with *in vitro* studies on the type I IFN response of normal human bronchial epithelial cells to RSV in conjunction with mAbs to either the F or G protein which showed clear superiority for targeting the G protein (48).

TLR3D3 is a native human mAb that binds the G protein CCD with low pM affinity; it has strong activities as both an antiviral and for immune response normalization (189). It is currently in IND-enabling preclinical development. In light of the accumulated results summarized here on the mechanisms underlying RSV disease, it is appropriate to test this agent as a post-infection treatment. If proven effective, design of a vaccine to induce comparable mAbs will benefit from recently published structural analysis of the binding of TRL3D3 to the G protein CCD (32).

## Conclusion

RSV infections continue to be a major cause of morbidity and mortality around the world affecting a wide variety of patients. Infants, the elderly, and those with comorbidities are at particularly high risk of hospitalization and death. Mainstream therapy remains restricted to supportive care. Despite successful antigen presentation leading to high titer of neutralizing antibodies by several approaches, we still do not have a licensed vaccine. Although the single licensed monoclonal antibody, palivizumab, is effective, it protects only a minor fraction of the population at high risk. Advances in therapeutic and vaccine development for RSV has mainly been hampered by the lack of understanding of the immune response to the virus both in the setting of primary infection as well as recurrent reinfections. Diverse approaches have converged over the last few years on identification of Type I IFN as a key actor and a readily measured biomarker of the broader innate immune response. Clinical studies in human infants have shown that RSV is a poor inducer of type I IFN responses, and there is accumulating literature reporting an inverse correlation between type I IFN responses and disease severity.

As our understanding improves of how viral proteins modify host immune responses, and the age dependence of those responses, research efforts can focus on development of effective countermeasures to overcome the virus's sophisticated sabotage of the host immune system. Animal models, complemented by studies on human cells *in vitro*, continue to be essential in the discovery and/or confirmation of the key features surrounding the host-virus interaction. Mouse models have proven to be particularly informative, including demonstrations that neonatal mice fail to produce IFN-α in the setting of RSV infection due to poor pDC recruitment, and that administration of IFNα decreases Th2-biased immunopathology and viral load. In addition, and importantly, administration of IFNα enhances mucosal RSV specific IgA production, which is critical given the clinical evidence that suggests that mucosal antibodies correlate better than systemic antibodies with protection. Although the known toxicities of recombinant IFN precludes use in this setting, a variety of approaches to restoring the normal IFN response have been identified, offering new opportunities for both therapeutic and vaccine discovery.

## Author Contributions

All authors listed have made a substantial, direct and intellectual contribution to the work, and approved it for publication.

### Conflict of Interest Statement

LK is an employee of Trellis Bioscience, and holds an equity interest in the company. The remaining authors declare that the research was conducted in the absence of any commercial or financial relationships that could be construed as a potential conflict of interest.
